# Investigation of Healthcare-Acquired Infections and Antimicrobial Resistance in an Italian Hematology Department before and during the COVID-19 Pandemic

**DOI:** 10.3390/microorganisms12071296

**Published:** 2024-06-26

**Authors:** Federica Petrone, Carmine Gizzi, Alessandro Andriani, Vincenza Martini, Roberta Sala, Angela Abballe, Lucia Capoccetta, Angela Spicciato, Marco Alfio Cutuli, Antonio Guarnieri, Noemi Venditti, Roberto Di Marco, Giulio Petronio Petronio

**Affiliations:** 1 Department of Medicina e Scienze della Salute “V. Tiberio”, Università degli Studi del Molise, 86100 Campobasso, Italy; federica.petrone@unimol.it (F.P.); angelaspicciato@gmail.com (A.S.); m.cutuli@studenti.unimol.it (M.A.C.); a.guarnieri@studenti.unimol.it (A.G.); noemi.venditti@responsible.hospital (N.V.); roberto.dimarco@unimol.it (R.D.M.); giulio.petroniopetronio@unimol.it (G.P.P.); 2 UOC Ematologia, Fabrizio Spaziani Hospital, 03100 Frosinone, Italy; alessandro.andriani1@tin.it (A.A.); vincenzamartini@inwind.it (V.M.); roberta.sala@aslfrosinone.it (R.S.); angela.abballe@aslfrosinone.it (A.A.); lucia.capoccetta@aslfrosinone.it (L.C.); 3 UO Laboratorio Analisi, Responsible Research Hospital, 86100 Campobasso, Italy

**Keywords:** healthcare-acquired infections, antimicrobial resistance, COVID-19 pandemic, microbial identification, antibiotic susceptibility, hematology

## Abstract

Background: The COVID-19 pandemic has made antibiotic resistance (AMR) and healthcare-acquired infections (HAIs) increasingly serious problems. Point-prevalence Surveys (PPS) and other surveillance techniques are essential for antimicrobial management and prevention. Methods: In a hematology department of an Italian hospital, the prevalence of HAI, microbiology, and AMR were examined in this retrospective study in two different periods, namely 2019 and 2021 (pre-pandemic and during the pandemic, respectively). Comparisons were made between patient demographics, hospitalization duration, surveillance swabs, and HAIs. Findings: There was no discernible variation in the prevalence of HAI between 2019 and 2021. Higher rates of HAI were connected with longer hospital stays. Variations in antimicrobial susceptibility and species distribution were found by microbiological analysis. Discussion: The incidence of HAI stayed constant during the epidemic. Nevertheless, shifts in antibiotic susceptibility and microbiological profiles highlight the necessity of continuous monitoring and care. Conclusions: Despite the difficulties of COVID-19, ongoing surveillance and infection control initiatives are crucial for halting HAIs and battling antimicrobial resistance (AMR) in healthcare environments. To fully understand the pandemic’s long-term impact on the spread of infectious diseases and antibiotic resistance, more research is required.

## 1. Introduction

Healthcare-acquired infections (HAIs) are adverse events that cause significant repercussions on system management, expenses [1,2,3], patients, visitors, and health workers [4]. Thus, the prolongation of hospitalization days [2,5,6,7], morbidity, disability, and mortality [2,8], together with antimicrobial resistance (AMR) onset and spread [9,10,11] are the most significant implications of HAIs. The World Health Organisation’s (WHO) 2011 report on the endemic burden of HAIs worldwide found that 7% of patients acquire at least one HAI during hospitalization in developed countries, with an increased rate from 10% to 15% in developing countries [8]. HAI prevalence rates are estimated to be between 5.7% and 19.1% in low- and middle-income countries, and between 5.7% and 7.5% in high-income countries [12]. Surveillance and control strategies are essential to prevent HAIs and deliver safe, quality care by reducing AMR [13,14]. Point-prevalence Surveys (PPS) are the most widely used strategy for HAI surveillance [15], and the most time- and cost-effective one, as they estimate the burden of HAIs and related risk factors [16,17]. The European Centre for Disease Prevention and Control (ECDC) coordinates all HAI surveillance, control, and prevention activities [18]. The ECDC’s first PPS was conducted in 2011–2012, and the point prevalence of HAIs in Europe was 6% [19]. Approximately 4.5 million people in Europe contract HAIs every year [20], with 37,000 deaths as a direct result of infection [21] and an extension of hospital bed days by more than 16 million [22]. AMR causes 426,277 cases of HAIs in Europe each year, resulting in over 33,000 deaths [11]. The COVID-19 pandemic has increased the risk of infectious disease transmission in the nosocomial setting. The SARS-CoV-2 coronavirus is mainly transmitted through droplets and aerosols [23]. Healthcare facilities and community centers are frequented by large numbers of inpatients or outpatients and visitors and are thus among the places at greatest risk for the spread of infections, including that of COVID-19 [24]. The WHO declared a state of pandemic in March 2020 [25]. Italy declared a state of emergency in January 2020, and regulations and legislation were implemented to contain the spread of COVID-19 [26]. The ECDC report in February 2020 reiterated the need for personal protective equipment (PPE) such as face masks, eye protection, gowns/suits, and gloves in healthcare settings [27]. The WHO published guidelines in June 2020 recommending PPE use in all care settings, including non-COVID-19 settings [28]. Considering the spread of the COVID-19 pandemic, the aim of this study is to investigate the incidence, microbiology, and corresponding AMR of care-related infections in patients admitted to the hematology department of an Italian hospital.

## 2. Materials and Methods

This was a retrospective observational study. The prevalence and epidemiology of HAI and AMR were evaluated by comparing patients’ medical records from 1 January to 31 December 2021 (pandemic) with those collected during the same period in 2019 (pre-pandemic). The study population consisted of dismissed patients who attended the Haematology Department of P.O.F. Spaziani Frosinone Italy during both the 2019 and 2020 time frames under examination. Patients diagnosed with COVID-19 were excluded from the study.

Data were collected from weekly surveillance swabs, performed in the hematology department, and classified according to collection site as hemoculture, pharyngeal, nasal, rectal, urinoculture, sputum, and coproculture. Moreover, additional information, such as age, gender, and admission diagnoses, found in the Hospital Discharge Form were taken into account. All records were collected anonymously and under current Italian privacy laws (Legislative Decree no.196 of 2003), with prior approval of the facility management.

### 2.1. Definition Criteria for HAIs

HAI patients and pre-HAIs were stratified from the study population according to the criteria proposed by the American Centers for Disease Control and Prevention (CDC) and the National Healthcare Safety Network (NHSN) [29], namely as the absence of ongoing infection in hospital entry developed at least 3 days after admission [30].

### 2.2. Microbial Identification and Antibiotic Susceptibility

Microbial identification and antibiograms were carried out by the microbiology laboratory of Fabrizio Spaziani Hospital of Frosinone, Italy, using VITEK^®^ (bioMérieux SA, Marcy l’Etoile, France) automatic platform. Microorganisms’ antibiotic susceptibility was expressed as MIC (µg/mL) according to Clinical Laboratory Standards Institute (CLSI) breakpoints [31].

### 2.3. Statistical Analysis

Statistical analysis was performed by IBM SPSS Statistics 26 software. Two-tailed *p* < 0.05 was considered statistically significant for all analyses. Categorical variables were summarized using frequencies and percentages; continuous variables were summarized using means and standard deviations since the distributions were symmetrical. For the univariate analysis, we used χ^2^ tests to compare categorical variables between the groups and the Student’s *t*-test for independent samples to compare continuous variables between the groups. HAI infection occurrence was modeled by mean-parametrized Conway–Maxwell (COM) Poisson regression for undispersed count data [32] by R studio 2022.02.1 software statistics.

## 3. Results

### 3.1. Study Population

In 2019, among the 174 patients who attended the hematology unit, 88 were men (50.6%) and 86 were women (49.4%). The mean age was 65.43 ± 13.6 years, with a median of 68.0 and a range of 20 to 92. On the other hand, in 2021, the number of patients attending the hematology unit was 159, of whom 84 (52.8%) were men and 75 (47.2%) were women. The mean age was 65.58 ± 15.0 years, with a median of 70.0 and a range of 18 to 92 (Table 1). Concerning hospitalization days: An average of 29.5 ± 28.7 with a median of 21.0 and a range of 2 to 191 days in 2019, towards an average of 23.2 ± 17.6 with a median of 18.0 and a range of 2 to 90 days in 2021 (Table 1). No significant differences were found for gender (*p* = 0.742), age (*p* = 0.532), hospitalization days (*p* = 0.195), and admission diagnosis among the two study groups (Table 1). Patients were tested for pharyngeal, nasal, and rectal sampling (98.3%), and coproculture (19.5%). Despite an increase in blood culture testing (+28.7%) and a decrease in coproculture (−12.6%) in 2021 compared to 2019, the number of overall tests did not change significantly among the two study periods (*p*-Value > 0.05) (Table 1).

### 3.2. Hospital-Acquired Infections and Microbiological Testing

HAI rates of the study population attending the hematology unit in 2019 and 2021 according to sampling profiles are shown in Table 2

No significant difference was found in the two groups: 38.5% of patients in 2019 and 37.7% in 2021 had an HAI (*p* = 0.910) (Table 2).

In 2019, among the 67 HAI patients who attended the hematology unit, 41 were men (61.2%) and 26 were women (38.8%). The mean age was 65.90 ± 11.3 years, with a median of 69.0 and a range of 41 to 87. On the other hand, in 2021 among the 60 HAI patients, 26 (43.3%) were men and 34 (56.6%) were women. The mean age was 65.92 ± 13.3 years, with a median of 67.0 and a range of 27 to 90 (Table 2).

Concerning the hospitalization days, a significant difference (*p* = 0.020) was found (Table 2). Indeed, an average of 29.5 ± 28.7 with a median of 21.0 and a range of 2 to 191 days in 2019, towards an average of 23.2 ± 17.6 with a median of 18.0 and a range of 2 to 90 days in 2021 (Table 2). No significant differences in gender (*p* = 0.052) and age (*p* = 0.992 among the two study groups were found (Table 2).

In 2019, almost all patients were tested for pharyngeal (97.0%), nasal (95.5%), and rectal sampling (97.0%), while only 4.5% for coproculture. Although there was a significant increase in the number of blood cultures (+16.4%) and a decrease in co-cultures (−20.9%) in 2021, the number of positive tests for all analyses did not change significantly in the two study periods (*p*-Value >0.05) (Table 2).

### 3.3. Mean-Parametrized Conway–Maxwell (COM) Poisson Regression [32]

The effect of hospitalization days, age, and gender of patients on the 2019 and 2021 HAI rate was studied by the COM Poisson regression for the under-dispersed count to model the occurrences of hospital infections using the hospitalization days numbers, age, and gender as predictors. According to the model’s outputs (Table 3), only the hospitalization days are closely correlated with the frequency of HAIs in 2019 (*p*-value 0.011). According to the results of the model (Table 3), in 2019, only days of hospitalization correlated closely with the frequency of HAIs (*p*-value 0.011), while none of the parameters correlated with the frequency of HAIs in 2021.

Moreover, each additional hospitalization day is associated with an increase in the HAI rate by a factor of 1.012 (Equation (1)). Alternatively, each additional hospitalization day is associated with an increased HAI occurrence of 1.2% (Equation (2)). Lastly, hospitalization days are associated with HAI occurrence, on average, of 1% to 2.2% (Equations (3) and (4)).

Equation (1): COM Rate Ratio of HAI for Hospitalization Days in 2019

eCoef hospitalization Days = e0.012 = 1.012(1)

Equation (2): % Rate Ratio

Rate Ratio − 1 × 100% = 1.012 − 1 × 100% = 1.2%(2)

Equation (3): 95% Confidence Interval

exp Rate Ratio ± 2 × SE = e^log^(0.012 ± 2 × 0.005) = [1.010, 1.022] (3)

Equation (4): 95% Confidence Interval Percentage

Confidence Interval − 1 × 100% = [1.010, 1.022] − 1 × 100% = [1, 2.2]%(4)

### 3.4. Microbiological Analysis of HAI

According to the isolation source, microorganism distribution was analyzed among HAI patients (Table 4). Although the total number of microorganisms isolated was not significantly different among the sample types (*p* > 0.05), differences were found in species distribution according to the isolation source. In pharyngeal samples, there was a 16.5% increase in *C. albicans* (*p* = 0.020) and a 20.6% decrease in *E. faecalis* (*p* = 0.012) in 2021. Likewise, rectal samples showed a 28.5% increase in *K. pneumoniae* (*p* = 0.009) and a 36.2% reduction in *E. coli* isolation (*p* = 0.019). Lastly, for urinocoltures, an increase in *E. faecalis* of 37.5% (*p* = 0.036) and a decrease in *E. coli* of 64.3% (*p* = 0.006) were found.

Finally, HAI antimicrobial susceptibility in the two study periods was analyzed. Only statistically significant results (*p* < 0.05) were reported in Table 5. The complete list of microorganisms and drugs tested can be retrieved from Supplementary Tables S1 and S2. Three microbial strains and one yeast exhibited notable changes in antimicrobial susceptibility (i.e., *S. haemolyticus*, *E. coli*, *E. faecalis*, and *C. albicans*). Regarding *S. haemolyticus*, an increase in linezolid-resistant strains alone (+81.8% *p* < 0.001) and a reduction for vancomycin and teicoplanin were observed (−49.3% *p* = 0.016 and −51.3% *p* = 0.015, respectively) (Table 5). The enhanced susceptibility to ampicillin penicillin and the second-generation cephalosporin cefuroxime in *E. coli* strains (−48.0% *p* = 0.001 and −22.8% *p* = 0.005) was opposed to the increased carbapenems resistance. The greatest increment was observed for ertapenem (+45.6% *p* = 0.004), followed by imipenem (+24.9% *p* = 0.040) and meropenem (+9.0% *p* = 0.017). Finally, improved susceptibility to the combination of Trimethoprim/Sulfamethoxazole (−36.3% *p* = 0.012) was also reported (Table 5). A similar trend for penicillin was also observed for *E. faecalis* with a reduction of 85.7% (*p* = 0.015) in the susceptible strains number; on the other hand, a notable drop in resistance towards the antifungals caspofungin (*p* = 0.030) and voriconazole (*p* = 0.048) was observed (Table 5).

## 4. Discussion

The pandemic has inevitably changed medical care practices, through the implementation of guidelines, protocols, and procedures, aimed at infection containment, not only in the hospital setting but also in the community [25,29], resulting in high stress for healthcare workers [33].

Although HAIs are linked to different health policy management across hospitals, our data about the Haematology Department at P.O.F. Spaziani Frosinone Italy reported in this study revealed no significant difference in the incidence of healthcare-acquired infections (HAIs) between the pre-pandemic and pandemic period (Table 2). Furthermore, as reported in the mean-parametrized Conway–Maxwell (COM) Poisson regression section, the incidence of HAIs was found to be correlated with the increase in hospitalization days. In 2019, days of hospitalization were found to be closely correlated with the incidence of HAIs (*p* = 0.011); specifically, each day of hospitalization is associated with a 1.2% increase in the frequency of HAIs. In 2021, there was an 8.62% reduction in hospitalizations and a statistically significant (*p* = 0.020) reduction in days of hospitalization (Table 3). On the other hand, epidemiological findings showed an increase in the isolation of *C. albicans* in pharyngeal swabs (*p* = 0.020), a reduction in *E. coli* (*p* = 0.019), and an increase in *K. pneumoniae* (*p* = 0.009) in rectal swabs. Additionally, *E. faecalis* isolates were found to be increasing in urine cultures (*p* = 0.036) and decreasing in pharyngeal swabs (*p* = 0.006) (Table 4).

Regarding antibiotic resistance (AMR), there was a decrease in sensitivity to Ampicillin (*p* = 0.001) and an increase in sensitivity to Carbapenems (Ertapenem *p* = 0.004; Imipenem *p* = 0.040; Meropenem *p* = 0.017) for *E. coli*. There was also a reduction in susceptibility of *E. faecalis* (*p* = 0.015) to Ampicillin and of *S. haemolyticus* to glycopeptides (Vancomycin *p* = 0.016; Teicoplanin *p* = 0.015) (Table 5).

The key findings of this study align with those from research conducted across various clinical settings. Data from the literature shows no significant difference in HAI incidence between the pre-pandemic and pandemic periods. A study by Tham et al. investigated the incidence of HAIs in patients admitted to a surgical ward at the Royal Melbourne Hospital (RMH), Melbourne, Victoria, Australia in 2019 and 2020 [34]. In the study by Mohammadi et al., data from the entire Sina Hospital in Iran for 9 months of 2019 and 2020 were compared, and, again, the data were unchanged [35]. Also, in the study by Lo et al. in a Taiwan medical center there was no statistically significant change in the incidence of HAIs between 2018, 2019, and 2020 [36].

To the best of the researcher’s knowledge, this study is the first to provide evidence for an increased rate of HAI for patients with longer hospitalization before and after the pandemic. There is strong evidence for this in the literature, due to the great social and economic costs of this problem. Indeed, hospitalization expenses and duration of stay as a result of HAI increased by 39.38% and 31.25%, respectively, according to multi-center research conducted in China by Lu et al. [6]. However, Hollenbeak et al. point out that it is challenging to directly compare the cost estimates due to variations in study populations, designs, and cost perspectives [37]. Furthermore, according to Scott et al., the anticipated societal benefits of preventing HAIs surpass the direct medical cost reductions when considering the societal cost perspective that encompasses long-term consequences, lost productivity, premature death, and other expenses [38].

Regarding admission rates, length of stay, and in-hospital mortality during the pandemic, a survey conducted in Qatar’s four large hospitals revealed a significant decrease in hospitalizations for most acute diseases during the COVID-19 pandemic, except for respiratory tract infections. The duration of hospitalizations decreased, but in-hospital deaths remained unchanged. This could impact intensive care patients requiring severe medical or surgical treatment [39].

Comparison with data from the literature of epidemiological findings regarding the distribution of isolated microorganisms requires certain considerations. These include the type of sample isolated and the characteristics of the study population, such as age, sex, lifestyle, and comorbidities. In this scenario, a previous study conducted by Petrone et al. on the epidemiology of diabetic foot ulcers (DFUs) highlighted that BMI and overweight are factors that expose diabetic individuals to an increased risk of infection [40].

Moreover, a point prevalence study conducted at the Azienda Ospedaliero-Universitaria of Sassari by Deiana et al. examined 655 patients across medical, surgical, and intensive care units during the pandemic. This study compared findings with a previous study conducted before the pandemic, revealing an increase in HAIs caused by *K. pneumoniae* and a decrease in those attributed to *E. coli*. [41]. Cornejo-Juárez et al. conducted a similar study in a Mexican cancer hospital, recording an increase in bacteremia from *E. coli*, *Stenotrophomonas maltophilia*, and *S. aureus* during the pandemic period [42]. Similar findings were reported by Ismaeil et al. in a retrospective cohort study from a tertiary hospital in Malaysia with a reduction of *E. faecalis* during the pandemic period [43].

Analogous considerations must also be made for microbial susceptibility results. Khoshbakht et al. in northeast Iran found an increase in AMR of *E. coli* over the period of 2020–2022 [44]. Langford et al.’s meta-analysis investigating the impact of the COVID-19 pandemic on antimicrobial resistance in all healthcare settings showed a possible increase in AMR in Gram - bacteria and no change in antimicrobial resistance in Gram + bacteria [45].

Although the pandemic has led to significant changes in medical care practices, according to the given pieces of evidence, there are no significant variations in the HAI rates. The susceptibility profile showed differences in the resistance of the microbial strains whereby some of them displayed higher levels of resistance to specific drugs, while others exhibited increased sensitivity. Altogether, these findings underscore the importance of continuing surveillance and antimicrobial stewardship programs in counteracting antimicrobial resistance with the emergence of COVID-19, emphasizing the importance of infection control measures regardless of environmental phenomena like the pandemic.

Some of the limitations of this study are related to its retrospective observational design; data were collected over 12 months in an Italian hospital and pertained only to patients receiving care at the hematology department. However, differences in data collection techniques and the delivery of healthcare as captured in the studies could pose a challenge to comparing our results with studies in the literature performed in different settings.

Therefore, future research and epidemiological surveillance should evaluate the possible changes in HAIs and antibiotic resistance rates after implementing pandemic-associated healthcare changes in different healthcare systems. It should also assess the impact of individual infection prevention and control measures put in place during the pandemic.

## 5. Conclusions

Despite the pandemic imposing unavoidable changes in healthcare practices, the rate of HAIs showed no contrastive trends between the pre-pandemic and the pandemic periods. Subsequent measures should therefore be implemented in conducting equally efficient surveillance efforts to prevent HAIs and antibiotic resistance in nosocomial settings. This study contributes to the understanding of how the interface of public health interventions, antibiotic consumption, and infectious diseases needs to be studied during the COVID-19 pandemic. Therefore, to quantify and qualify the high risk of additional infections and antibiotic resistance due to actions undertaken during a pandemic, further research studies are required. Also, it is emphasized that prospective studies and awareness-raising activities are needed to decrease HAIs and antibiotic resistance in healthcare institutions.

## Figures and Tables

**Table 1:** Patients attending the hematology unit in 2019 and 2021 and microbiological tests for the study population.

	2019 N (%)	2021 N (%)	Change %	*p*-Value
Total	174	159		
GENDER				
Man	88 (50.6)	84 (52.8)		0.742
Woman	86 (49.4)	75 (47.2)		
AGE				0.532
Mean ± D.S.	65.4 ± 13.6	65.6 ± 15.0		
Median	68.0	70.0		
Minimum	20	18		
Maximum	92	92		
HOSPITALIZATION DAYS				0.195
Mean ± D.S.	29.5 ± 28.7	23.2 ± 17.6		
Median	21.0	18.0		
Minimum	2	2		
Maximum	191	90		
ADMISSION DIAGNOSIS				
Oncohematological diseases	106 (60.9)	87 (54.7)		0.268
*Reticulosarcoma lymph nodes multiple sites*	22 (12.6)	5 (3.1)		**0.002**
Hematological diseases (non-oncological)	20 (11.5)	27 (17)		0.160
Cardiac diseases	1 (0.6)	1 (0.6)		1.000
Respiratory diseases	2 (1.1)	1 (0.6)		1.000
Infections	31 (17.8)	32 (22)		0.409
Renal diseases	3 (1.7)	2 (1.3)		1.000
Other	11 (6.3)	6 (3.8)		0.328
EMOCULTURE, n (%)	123 (70.7)	157 (99.4)	+28.7	**<0.001**
Positive	22 (17.9)	37 (23.6)		0.302
PHARYNGEAL SAMPLING, n (%)	171 (98.3)	158 (99.4)		0.624
Positive	49 (28.7)	50 (31.6)		0.631
NASAL SAMPLING, n (%)	171 (98.3)	158 (99.4)		0.624
Positive	29 (17.0)	31 (19.6)		0.569
RECTAL SAMPLING, n (%)	171 (98.3)	158 (99.4)		0.624
Positive	54 (31.6)	35 (22.3)		0.063
URINOCULTURE, n (%)	69 (39.7)	54 (34.0)		0.303
Positive	18 (26.1)	9 (16.7)		0.274
SPUTUM SAMPLING, n (%)	7 (4.0)	2 (1.3)		0.178
Positive	3 (42.9)	1 (50.0)		1.000
COPROCULTURE, n (%)	34 (19.5)	11 (6.9)	−12.6	**0.001**
Positive	0	0		-

Positive: pathogenic microorganisms’ isolation. The change in percentages (Change %) has been calculated for statistically significant results only (*p* < 0.05).

**Table 2:** HAI rates of study population attending the hematology unit in 2019 and 2021 according to sampling profiles.

	2019 N = 174	2021 N = 159	Change %	*p*-Value
HAI, n (%)	67 (38.5)	60 (37.7)		0.910
GENDER, n (%)				0.052
Man	41 (61.2)	26 (43.3)		
Woman	26 (38.8)	34 (56.6)		
AGE (%)				0.992
Mean ± D.S.	65.90 ± 11.3	65.92 ± 13.3		
Median	69.0	67.0		
Minimum	41	27		
Maximum	87	90		
HOSPITALIZATION DAYS (%)				**0.020**
Mean ± D.S.	46.5 ± 30.7	35.7 ± 19.1		
Median	41.0	32.5		
Minimum	4	5		
Maximum	181	90		
EMOCULTURE, n (%)	56 (83.6)	60 (100.0)	+16.4	**0.001**
Positive	16 (28.6)	25 (41.7)		0.100
PHARYNGEAL SAMPLING, n (%)	65 (97.0)	60 (100.0)		0.498
Positive	31 (47.7)	27 (45.0)		0.487
NASAL SAMPLING, n (%)	64 (95.5)	60 (100.0)		0.246
Positive	13 (20.3)	8 (13.3)		0.345
RECTAL SAMPLING, n (%)	65 (97.0)	60 (100.0)		0.498
Positive	29 (44.6)	23 (38.3)		0.586
URINOCULTURE, n (%)	43 (64.2)	26 (43.3)	−20.9	**0.021**
Positive	14 (32.6)	8 (30.8)		1.000
SPUTUM SAMPLING, n (%)	3 (4.5)	1 (1.7)		0.621
Positive	3 (100.0)	1 (100.0)		-

Positive: isolation of pathogenic microorganisms. Percent change (Change %) was calculated only for statistically significant results (*p* < 0.05).

**Table 3:** COM Poisson regression analysis for 2019 and 2021.

2019	Coefficient (Coef)	Standard Error (SE)	*p*-Value
Hospitalization days	0.012	0.005	**0.011**
Age	0.0007	0.0137	0.958
Gender	−0.1628	0.3170	0.6075
Intercept	1.085	0.179	1.412 × 10^−9^
2021			
Hospitalization days	0.008	0.007	0.291
Age	0.008	0.011	0.457
Gender	−0.2115	0.2835	0.456
Intercept	0.9238	0.193	1.737 × 10^−6^

**Table 4:** HAI microorganisms distribution in positive samples according to the isolation source.

	Positive Samples		Change %	*p*-Value
	2019	2021		
EMOCULTURE				
*Escherichia coli*	4 (25.0)	2 (6.6)		0.187
*Staphylococcus hominis*	1 (6.3)	6 (20.0)		0.215
*Srteptococcus epidermidis*	2 (12.5)	6 (20.0)		0.448
*Staphylococcus haemolyticus*	1 (6.3)	4 (13.3)		0.632
*Candida albicans*	0 (0.0)	1 (3.3)		1.000
*Candida glabrata*	0 (0.0)	1 (3.3)		1.000
*Listeria monocytogenes*	0 (0.0)	1 (3.3)		1.000
*Klebsiella pneumoniae*	0 (0.0)	1 (3.3)		1.000
*Enterobacter cloacae*	1 (6.3)	0 (0.0)		0.390
*Enterococcus faecium*	3 (18.8)	1 (3.3)		0.281
*Staphylococcus caprae*	0 (0.0)	1 (3.3)		1.000
*Streptococcus agalactiae*	0 (0.0)	1 (3.3)		1.000
*Staphylococcus aureus*	1 (5.0)	1 (3.3)		1.000
*Acinetobacter baumannii*	1 (6.3)	0 (0.0)		0.390
*Aeromonas veronii biovar veronii*	1 (6.3)	0 (0.0)		0.390
*Klebsiella variicola*	1 (6.3)	0 (0.0)		0.390
*Enterococcus faecalis*	0 (0.0)	1 (3.3)		1.000
*Streptococcus gallolyticus*	0 (0.0)	1 (3.3)		1.000
*Enterococcus casseliflavus*	0 (0.0)	1 (3.3)		1.000
*Candida tropicalis*	0 (0.0)	1 (3.3)		1.000
Total number of microorganisms, N (%)	16 (100)	30 (100)		0.083
PHARYNGEAL SAMPLING				
*Klebsiella pneumoniae*	1 (2.9)	3 (8.3)		0.329
*Escherichia coli*	0 (0.0)	2 (5.5)		0.212
*Candida albicans*	1 (2.9)	7 (19.4)	+16.5	**0.020**
*Candida glabrata*	2 (5.9)	3 (8.3)		0.656
*Citrobacter koseri*	0 (0.0)	1 (2.8)		0.466
*Staphylococcus haemolyticus*	9 (26.5)	7 (19.4)		1.000
*Staphylococcus aureus*	7 (20.6)	0 (0.0)		0.057
*Candida tropicalis*	0 (0.0)	2 (5.5)		0.212
*Enterobacter cloacae*	1 (2.9)	1 (2.8)		1.000
*Enterococcus faecalis*	7 (20.6)	0 (0.0)	−20.6	**0.012**
*Streptococcus pneumoniae*	1 (2.9)	0 (0.0)		1.000
*Enterococcus faecium*	1 (2.9)	2 (5.5)		0.593
*Pseudomonas aeruginosa*	1 (2.9)	3 (8.3)		0.329
*Candida norvegensis*	1 (2.9)	0 (0.0)		1.000
*Enterobacter asburiae*	0 (0.0)	1 (2.8)		0.466
*Stenotrophomonas maltophilia*	0 (0.0)	2 (5.5)		0.212
*Serratia marcescens*	1 (2.9)	0 (0.0)		1.000
*Staphylococcus lugdunensis*	1 (2.9)	0 (0.0)		1.000
*Candida krusei*	0 (0.0)	1 (2.8)		0.466
*Enterobacter kobei*	0 (0.0)	1 (2.8)		0.466
*Pseudomonas cactophila*	0 (0.0)	1 (2.8)		0.466
Total number of microorganisms, N (%)	34	36		0.287
NASAL SAMPLING				
*Staphylococcus aureus*	8 (57.1)	3 (42.8)		0.387
*Staphylococcus haemolyticus*	0 (0.0)	2 (28.6)		0.133
*Candida glabrata*	0 (0.0)	1 (14.3)		0.381
*Enterobacter cloacae*	1 (7.1)	0 (0.0)		1.000
*Escherichia coli*	1 (7.1)	0 (0.0)		1.000
*Staphylococcus lugdunensis*	0 (0.0)	1 (14.3)		0.381
*Klebsiella pneumoniae*	1 (7.1)	0 (0.0)		1.000
*Stenotrophomonas maltophilia*	1 (7.1)	0 (0.0)		1.000
*Staphylococcus pseudintermedius*	1 (7.1)	0 (0.0)		1.000
*Serratia marcescens*	1 (7.1)	0 (0.0)		1.000
Total number of microorganisms, N (%)	14	7		0.436
RECTAL SAMPLING, N (%)				
*Escherichia coli*	27 (90.0)	14 (53.8)	−36.2	**0.019**
*Klebsiella pneumoniae*	3 (10.0)	10 (38.5)	+28.5	**0.009**
*Pseudomonas aeruginosa*	0 (0.0)	1 (3.8)		0.434
*Citrobacter freundii*	0 (0.0)	1 (3.8)		0.434
Total number of microorganisms, N (%)	30	26		0.686
URINOCOLTURE, N (%)				
*Enterococcus faecalis*	0 (0.0)	3 (37.5)	+37.5	**0.036**
*Candida albicans*	0 (0.0)	1 (12.5)		0.364
*Escherichia coli*	9 (64.3)	0 (0.0)	−64.3	**0.006**
*Klebsiella* *pneumoniae*	0 (0.0)	1 (12.5)		0.364
*Klebsiella* *oxytoca*	0 (0.0)	1 (12.5)		0.364
*Acinetobacter baumanii*	1 (7.1)	0 (0.0)		1.000
*Pseudomonas aeruginosa*	2 (14.3)	0 (0.0)		0.515
*Enterococcus* *cloacae*	1 (7.1)	0 (0.0)		1.000
*Enterococcus* *faecium*	1 (7.1)	0 (0.0)		1.000
*Staphylococcus* *aureus*	0 (0.0)	1 (12.5)		0.364
*Citrobscter freundii*	0 (0.0)	1 (12.5)		0.364
Total number of microorganisms, N (%)	14	8		0.898
SPUTUM SAMPLING, N (%)				
*Staphylococcus haemoliticus*	1 (33.3)	1 (100.0)		1.000
*Acinetobacter baumanii*	1 (33.3)	0 (0.0)		1.000
*Candida albicans*	1 (33.3)	0 (0.0)		1.000
Total number of microorganisms, N (%)	3	1		0.200

The change in percentages (Change %) has been calculated for statistically significant results only (*p* < 0.05).

**Table 5:** HAI antimicrobial susceptibility. S: susceptible; I: intermediate; R: resistant.

	2019 N (%)	2021 N (%)	Change %	*p*-Value
*Staphylococcus haemolyticus*	11 (100)	14 (100)		
Vancomycin				
S	4 (36.4)	12 (85.7)	−49.3	**0.016**
R	7 (63.6)	2 (14.3)		
Teicoplanin				
S	3 (27.3)	11 (78.6)	−51.3	**0.015**
R	8 (72.7)	3(21.4)		
Linezolid				
S	9 (81.8)	0 (0.0)	+81.8	**<0.001**
R	2 (18.2)	14 (100.0)		
*Escherichia coli*	41 (100)	18 (100)		
Ampicillin				
S	0 (0.0)	5 (27.8)	−48.0	**0.001**
I	4 (9.8)	0 (0.0)		
R	37 (74.0)	13 (26.0)		
Cefuroxime				
S	18 (43.9)	8 (44.4)	−22.8	**0.005**
I	0	4 (22.2)		
R	23 (56.1)	6 (33.3)		
Ertapenem				
S	20 (48.8)	1 (5.6)	+45.6	**0.004**
I	1 (2.4)	0 (0.0)		
R	20 (48.8)	17 (94.4)		
Imipenem				
S	25 (61.0)	10 (55.6)	+24.9	**0.040**
I	8 (19.5)	0 (0.0)		
R	8 (19.5)	8 (44.4)		
Meropenem				
S	21 (53.8)	4 (22.2)	+9.0	**0.017**
I	2 (5.1)	5 (27.8)		
R	16 (41.0)	9 (50.0)		
Trimethoprim/Sulfamethoxazole				
S	17 (41.5)	14 (77.8)	−36.3	**0.012**
R	24 (58.5)	4 (22.2)		
*Enterococcus faecalis*	7 (100)	4 (100)		
Ampicillin				
S	1 (14.3)	4 (100.0)	−85.7	**0.015**
R	6 (85.7)	0 (0.0)		
*Candida glabrata*	2 (100)	5 (100)		
Caspofungin				
S	1 (50.0)	0 (0.0)	+100	**0.030**
I	1 (50.0)	0 (0.0)		
R	0 (0.0)	5 (100.0)		
Voriconazole				
S	0 (0.0)	5 (100.0)	−100	**0.048**
I	2 (100.0)	0 (0.0)		

Only significative results (*p* < 0.050) have been reported. The change in percentages has been calculated for resistant strains (R change %).

## Data Availability

The original contributions presented in the study are included in the article/Supplementary Materials, further inquiries can be directed to the corresponding author/s.

## Supplementary Materials

The following supporting information can be downloaded at: https://www.mdpi.com/article/10.3390/microorganisms12071296/s1, Table S1: complete list of microorganisms and drugs tested for 2019; Table S2: complete list of microorganisms and drugs tested for 2021.
