# Supplementary material for: Investigation of Healthcare-Acquired Infections and Antimicrobial Resistance in an Italian Hematology Department before and during the COVID-19 Pandemic
Source: Microorganisms. 2024 Jun 26;12(7):1296. doi: 10.3390/microorganisms12071296 (PMC11278871; doi:10.3390/microorganisms12071296)
Supplement: Supplementary file 1 [file microorganisms-12-01296-s001.zip › microorganisms-3062070-supplementary.pdf]

Table S1 Complete list of microorganisms and drugs tested for 2019

MIC (µg/mL) Breakpoints		*Escherichia coli* n (%) = **41(37)**					*Staphylococcus aureus* n (%) = **16(15)**			*Staphylococcus haemolyticus* n (%) = **11(10)**			*Enterococcus faecalis* n (%) = **7(6)**
		EMO	TOF	NAS	TEK	URI	EMO	TOF	NAS	EMO	TOF	ESP	TOF
Ampicillin	S susceptible				2	2							6
	I intermediate												
	R resistant	4		1	25	7		3	1				1
	MIC 50	>8		>8	>8	>8		>1	>1				≤2
	MIC 90	>8		>8	>8	>8		>1	>1				4
Oxacillin	S susceptible							1	4				
	I intermediate												
	R resistant						1	6	4	1	9	1	
	MIC 50						>2	>2	0.5	>2	>2	>2	
	MIC 90						>2	>2	>2	>2	>2	>2	
Penicillin	S susceptible												
	I intermediate												
	R resistant						1	6	6				
	MIC 50						>0.25	>0.25	>0.25				
	MIC 90						>0.25	>0.25	>0.25				
Piperacillin	S susceptible				4								
	I intermediate												
	R resistant	4		1	23	1							
	MIC 50	>16		16	>16	>16							
	MIC 90	>16		16	>16	>16							
Piperacillin-	S susceptible			1	18	8							
	I intermediate	1											
	R resistant	3			9	1							
	MIC 50	≤1		4	≤1	≤1							
	MIC 90	4		4	>4	2							
Amoxicillin-	S susceptible	1			6	3							
	I intermediate			1									
	R resistant	3			12	2							
	MIC 50	8		8	>16	4							
	MIC 90	>16		8	>16	>16							
Cefalexine	S susceptible					3							
	I intermediate												
	R resistant					6							
	MIC 50					>16							
	MIC 90					>16							

MIC (µg/mL) Breakpoints		*Klebsiella pneumoniae* n (%) = **5(5)**			*Enterococcus faecium* n (%) = **5(5)**			*Enterobacter cloacae* n (%) = **4(4)**				*Acinetobacter baumanii* n (%) = **3(3)**			*Pseudomonas aeruginosa* n (%) = 3**(3)**	
		TOF	NAS	TEK	EMO	TOF	URI	EMO	TOF	NAS	URI	EMO	URI	ESP	TOF	URI
Ampicillin	S susceptible						1									
	I intermediate															
	R resistant	1	1	3	3			1	1	1	1	1	1	1		2
	MIC 50	>8	>8	>8	>8		≤2	>8	>8	>8	>8	>8	>8	>8		>8
	MIC 90	>8	>8	>8	>8		≤2	>8	>8	>8	>8	>8	>8	>8		>8
Oxacillin	S susceptible															
	I intermediate															
	R resistant															
	MIC 50															
	MIC 90															
Penicillin	S susceptible															
	I intermediate															
	R resistant				3											
	MIC 50				>0.25											
	MIC 90				>0.25											
Piperacillin	S susceptible			1					1						1	
	I intermediate			1												
	R resistant	1	1	1				1		1						
	MIC 50	>16	>16	>16				>16	≤4	>16					≤4	
	MIC 90	>16	>16	>16				>16	≤4	>16					≤4	
Piperacillin-	S susceptible								1		1				1	2
	I intermediate			1												
	R resistant	1	1	2				1		1						
	MIC 50	>4	>4	>4				>4	≤1	>4	≤1				≤1	≤1
	MIC 90	>4	>4	>4				>4	≤1	>4	≤1				≤1	≤1
Amoxicillin-	S susceptible															
	I intermediate															
	R resistant	1	1	3				1	1	1	1	1	1	1		2
	MIC 50	>16	>16	>16				>16	>16	>16	>16	>16	>16	>16		>16
	MIC 90	>16	>16	>16				>16	>16	>16	>16	>16	>16	>16		>16
Cefalexine	S susceptible															
	I intermediate															
	R resistant										1	1	1			2
	MIC 50										>16	>16	>16			>16
	MIC 90										>16	>16	>16			>16

MIC (µg/mL) Breakpoints		*Staphylococcus epidermidis* n (%) = **2(2)**	*Candida albicans* n (%) = **2(2)**		*Candida glabrata* n (%) = **2(2)**	*Candida norvegensis* n (%) = **1(1)**	*Staphylococcus hominis* n (%) = **1(1)**	*Aeromonas veroni bv veronii* n (%) = **1(1)**	*Klebsiella variicola* n (%) = **1(1)**	*Serratia marcescens* n (%) = **1(1)**	*Staphylococcus lugdunensis* n (%) = **1(1)**	*Streptococcus pneumoniae* n (%) = **1(1)**	*Staphylococcus pseudintermedius* n (%) = **1(1)**	*Stenotrophomonas maltophilia* n (%) = **1(1)**
		EMO	TOF	ESP	TOF	TOF	EMO	EMO	EMO	TOF	TOF	TOF	NAS	NAS
Ampicillin	S susceptible								1					
	I intermediate													
	R resistant									1				1
	MIC 50								8	>8				>8
	MIC 90								8	>8				>8
Oxacillin	S susceptible										1		1	
	I intermediate													
	R resistant	2					2							
	MIC 50	>2					>2				≤0.25		≤0.25	
	MIC 90	>2					>2				≤0.25		≤0.25	
Penicillin	S susceptible													
	I intermediate													
	R resistant	2										1		
	MIC 50	>0.25										4		
	MIC 90	>0.25										4		
Piperacillin	S susceptible								1					
	I intermediate													
	R resistant									1				1
	MIC 50								≤4	>16				>16
	MIC 90								≤4	>16				>16
Piperacillin-	S susceptible								1					
	I intermediate													
	R resistant									1				1
	MIC 50								≤1	>4				>4
	MIC 90								≤1	>4				>4
Amoxicillin-	S susceptible								1					
	I intermediate													
	R resistant									1				1
	MIC 50								2	>16				>16
	MIC 90								2	>16				>16
Cefalexine	S susceptible													
	I intermediate													
	R resistant													
	MIC 50													
	MIC 90													

**Cefuroxim**	$S^{susceptible}$	2			12	3																														1					
	$I^{intermediate}$																																								
	$R^{resistant}$	2		1	15	6								1	1	3								1	1	1	1	2											1		1
	**MIC 50**	4		>8	>8	>8								>8	>8	>8								>8	>8	>8	>8	>8								4			>2		>8
	**MIC 90**	>8		>8	>8	>8								>8	>8	>8								>8	>8	>8	>8	>8								4			>2		>8
**Cefoxitine**	$S^{susceptible}$																																								
	$I^{intermediate}$																																								
	$R^{resistant}$												5				3	1	1																						
	**MIC 50**												>8				>8	>8	>8																						
	**MIC 90**												>8				>8	>8	>8																						
**Ceftazidime**	$S^{susceptible}$	3		1	16	4										1					1		1												1	1	1				
	$I^{intermediate}$					2																																			
	$R^{resistant}$	1			11	3								1	1	2				1		1					1	2													
	**MIC 50**	≤0.5		≤0.5	≤0.5	4								>8	>8	>8				>8	≤0.5	>8	≤0.5				>8	>8							≤0.5	≤0.5	≤0.5				
	**MIC 90**	>8		≤0.5	>8	8								>8	>8	>8				>8	≤0.5	>8	≤0.5				>8	>8							≤0.5	≤0.5	≤0.5				
**Cefotaxime**	$S^{susceptible}$	3		1	17	4										1					1		1													1	1				
	$I^{intermediate}$															1																									
	$R^{resistant}$	1			10	5								1	1	1				1		1		1	1	1	1	2											1		1
	**MIC 50**	≤1		≤1	≤1	>1								>4	>4	2				>4	≤1	>4	≤1	>4	>4	>4	>4	>4								≤1	≤1		>2		>4
	**MIC 90**	>4		≤1	>4	>4								>4	>4	>4				>4	≤1	>4	≤1	>4	>4	>4	>4	>4								≤1	≤1		>2		>4
**Cefixime**	$S^{susceptible}$					3																																			
	$I^{intermediate}$																																								
	$R^{resistant}$				8	6																	1																		
	**MIC 50**					>2																	>2																		
	**MIC 90**					>2																	>2																		
**Cefepime**	$S^{susceptible}$	3		1	12	3										1					1							1							1	1					
	$I^{intermediate}$					2																	1																		
	$R^{resistant}$	1			15	4								1	1	2				1		1					1	1									1		1		
	**MIC 50**	≤1		≤1	>8	4								>8	>8	>8				>8	≤1	>8	4				>8	8							≤1	≤1	>8		>2		
	**MIC 90**	>8		≤1	>8	>8								>8	>8	>8				>8	≤1	>8	4				>8	>8							≤1	≤1	>8		>2		
**Ceftaroline**	$S^{susceptible}$						1	2	6																													1			
	$I^{intermediate}$																																								
	$R^{resistant}$																3	1	1																						
	**MIC 50**						1	0.5	0.25								>1	>1	>1																			0.25			
	**MIC 90**						1	1	1								>1	>1	>1																			0.25			
**Ertapenem**	$S^{susceptible}$	3		1	9	7										1				1	1	1														1	1				
	$I^{intermediate}$																																								
	$R^{resistant}$	1			18	1								1	1	2							1	1	1	1	1	2													
	**MIC 50**	≤0.25		≤0.25	>1	≤0.25								>1	>1	>1				≤0.25	≤0.25	≤0.25	>1	>1	>1	>1	>1	>1								≤0.25	≤0.25				
	**MIC 90**	>1		≤0.25	>1	0.5								>1	>1	>1				≤0.25	≤0.25	≤0.25	>1	>1	>1	>1	>1	>1								≤0.25	≤0.25				
**Imipenem**	$S^{susceptible}$	4		1	12	8		1	1				6						1	1	1	1					1	1								1	1				
	$I^{intermediate}$				7	1										1							1																		
	$R^{resistant}$				8			2	1	1	4		1	1	1	2	3	1						1	1	1		1						1							1
	**MIC 50**	≤0.25		≤0.25	4	≤0.25		>8	≤2	>8	≤2		≤2	>8	>8	>8	>8	>8	≤2	≤0.25	1	≤0.25	8	>8	>8	>8	≤0.25	2						≤2		0.5	≤0.25				>8
	**MIC 90**	≤0.25		≤0.25	>8	2		>8	>8	>8	>8		4	>8	>8	>8	>8	>8	≤2	≤0.25	1	≤0.25	8	>8	>8	>8	≤0.25	>8						≤2		0.5	≤0.25				>8
**Meropenem**	$S^{susceptible}$	4		1	7	9														1	1	1	1					1								1	1				
	$I^{intermediate}$				2																							1													
	$R^{resistant}$				16									1	1	3								1	1	1	1												1		1
	**MIC 50**	≤0.125		≤0.125	>8	≤0.125								>8	>8	>8				≤0.125	≤0.125	≤0.125	≤0.125	>8	>8	>8	>8	0.5								≤0.125	≤0.125		>2		>8
	**MIC 90**	0.25		≤0.125	>8	0.25								>8	>8	>8				≤0.125	≤0.125	≤0.125	≤0.125	>8	>8	>8	>8	4								≤0.125	≤0.125		>2		>8
**Vancomicin**	$S^{susceptible}$						1	6	8		4		7				1												1					1				1	1	1	
	$I^{intermediate}$																																								
	$R^{resistant}$							1		1	5	1					2	1	1										1												
	**MIC 50**						≤0.5	1	1	>8	>8	>8	1				>8	>8	>8										≤0.5					≤0.5				≤0.5	2	≤0.5	
	**MIC 90**						≤0.5	2	1	>8	>8	>8	1				>8	>8	>8										8					≤0.5				≤0.5	2	≤0.5	

Fosfomycin	S susceptible	4		1	7	6	1	5	7		5					1				1	1	1	1						2						1				1	
	I intermediate					1																																		
	R resistant				20	2		2	1	1	4	1		1	1	2								1	1	1							1			1	1			1
	MIC 50	≤16		≤16	≤16	≤16	≤16	≤16	≤16	>64	32	>64		>64	>64	64				32	≤16	32	≤16	>64	>64	64			≤16				64		32	>64	>64		≤16	>64
	MIC 90	≤16		≤16	64	>34	≤16	64	≤16	>64	>64	>64		>64	>64	>64				32	≤16	32	≤16	>64	>64	64			32				64		32	>64	>64		≤16	>64
Daptomycin	S susceptible						1	5	8	1	8	1																	2				1				1		1	
	I intermediate																																							
	R resistant							2			1																													
	MIC 50						≤0.5	≤0.5	≤0.5	≤0.5	≤0.5	1																	≤0.5				≤0.5				≤0.5		≤0.5	
	MIC 90						≤0.5	2	≤0.5	≤0.5	1	1																	≤0.5				≤0.5				≤0.5		≤0.5	
Teicoplanin	S susceptible						1	7	8		3		6				1		1										1				1				1	1	1	
	I intermediate																																							
	R resistant									1	6	1	1				2	1											1											
	MIC 50						≤0.5	≤0.5	≤0.5	>8	>8	>8	≤0.5				>8	>8	≤0.5										≤2				≤0.5				1	≤1	≤0.5	
	MIC 90						≤0.5	≤0.5	≤0.5	>8	>8	>8	≤0.5				>8	>8	≤0.5										>8				≤0.5				1	≤1	≤0.5	
Colistin	S susceptible																																							
	I intermediate																																							
	R resistant				8											2										1	1									1				
	MIC 50				>4											>4										>4	>4									>4				
	MIC 90				>4											>4										>4	>4									>4				
Linezolid	S susceptible						1	7	8	1	7	1	7				2	1	1										1				1				1	1	1	
	I intermediate																																							
	R resistant										2						1												1											
	MIC 50						1	2	2	1	1	1	1				4	2	2										1				1				1	≤0.5	1	
	MIC 90						1	2	2	1	>4	1	1				>4	2	2										>4				1				1	≤0.5	1	
Cloramphenic	S susceptible																																							
	I intermediate																																							
	R resistant																																					1		
	MIC 50																																					>8		
	MIC 90																																					>8		
Erythromycin	S susceptible						1	5	4	1																			1								1		1	
	I intermediate																												1											
	R resistant							2	4		9	1	7				3	1	1														1					1		
	MIC 50						≤0.25	≤0.25	0.5	≤0.25	>2	>2	>2				>2	>2	>2										≤0.25				>2				0.5	>0.5	≤0.25	
	MIC 90						≤0.25	>2	>2	≤0.25	>2	>2	>2				>2	>2	>2										2				>2				0.5	>0.5	≤0.25	
Clindamycin	S susceptible						1	2	4	1																			1								1		1	
	I intermediate																																							
	R resistant							5	4		9	1	7																1				1					1		
	MIC 50						≤0.25	≤0.25	≤0.25	≤0.25	>1	>1	>1																≤0.25				≤0.25				≤0.25	>0.5	≤0.25	
	MIC 90						≤0.25	1	>1	≤0.25	>1	>1	>1																>1				≤0.25				≤0.25	>0.5	≤0.25	
Amikacin	S susceptible	3			22									1	1	3				1	1	1					1								1	1				
	I intermediate	1		1	5	1																																		
	R resistant																							1	1	1														1
	MIC 50	≤4		8	≤4	8								≤4	≤4	≤4				8	≤4	≤4		>16	>16	>16	≤4								≤4	8				16
	MIC 90	8		8	8	8								≤4	≤4	≤4				8	≤4	≤4		>16	>16	>16	≤4								≤4	8				16
Gentamicin	S susceptible	4		1	23	7	1	2	3		1					2					1		1				1	1							1		1		1	
	I intermediate																																							
	R resistant				4	2		5	5	1	8	1	5	1	1	1	3	1	1	1		1		1	1	1		1	2				1			1				1
	MIC 50	≤1		2	2	≤1	≤1	>4	≤1	>4	>4	>4	>4	>4	>4	≤1	4	>4	>4	>4	≤1	>4	≤1	>4	>4	>4	2	2	>4				4		≤1	4	≤1		≤1	4
	MIC 90	2		2	>4	>4	≤1	>4	>4	>4	>4	>4	>4	>4	>4	>4	>4	>4	>4	>4	≤1	>4	≤1	>4	>4	>4	2	>4	>4				4		≤1	4	≤1		≤1	4
Tobramycin	S susceptible	3			18	6										2					1		1				1								1	1				
	I intermediate																																							
	R resistant	1		1	9	3								1	1	1				1		1		1	1	1		2												1
	MIC 50	≤1		>4	2	≤1								≤1	>4	≤1				>4	≤1	>4	≤1	>4	>4	>4	2	>4							≤1	≤1				4
	MIC 90	>4		>4	>4	>4								>4	>4	>4				>4	≤1	>4	≤1	>4	>4	>4	2	>4							≤1	≤1				4

Tetracycline	S susceptible						1	6	5	1	1																		1				1				1		1	
	I intermediate										1																													
	R resistant							1	3		7	1																	1									1		
	MIC 50						≤0.5	≤0.5	≤0.5	≤0.5	>2	>2																	≤0.5				≤0.5				≤0.5	>4	≤0.5	
	MIC 90						≤0.5	≤0.5	>2	≤0.5	>2	>2																	>2				≤0.5				≤0.5	>4	≤0.5	
Tigecycline	S susceptible	3		1	23	8	1	7	7	1	6		7							1	1	1	1						1				1		1		1		1	
	I intermediate	1			2											1																				1				
	R resistant				2	1			1		3	1		1	1	2												2	1											
	MIC 50	≤0.5		≤0.5	≤0.5	≤0.5	≤0.25	≤0.25	≤0.25	≤0.25	0.5	>1	≤0.25	>2	>2	>2				≤0.5	1	≤0.5	≤0.5					>2	≤0.25				≤0.25		1	2	≤0.25		≤0.25	
	MIC 90	>2		≤0.5	1	1	≤0.25	≤0.25	≤0.25	≤0.25	>1	>1	≤0.25	>2	>2	>2				≤0.5	1	≤0.5	≤0.5					>2	1				≤0.25		1	2	≤0.25		≤0.25	
Rifampicin	S susceptible																																							
	I intermediate									1																														
	R resistant							3			7	1																	1											
	MIC 50							>1		0.5	>1	1																	1											
	MIC 90							>1		0.5	>1	1																	1											
Trimethoprim-	S susceptible	2		1	13	1	1	1	8	1	1					2					1		1						1				1	1	1		1	1	1	
	I intermediate																																							
	R resistant	2			14	8		6			8	1	7	1	1	1	2		1	1		1		1	1	1			1							1				1
	MIC 50	≤0.05		≤0.05	>0.05	>0.05	≤0.05	≤1/19	≤1/19	≤0.05	>0.05	>0.05	>0.05	>0.05	>0.05	≤0.05	≤0.05		>0.05	>0.05	≤0.05	>0.05	0.05	>0.05	>0.05	>0.05			≤0.05				0.05	≤0.05	≤0.05	>0.05	≤0.05	≤0.05	≤0.05	>0.05
	MIC 90	>0.05		≤0.05	>0.05	>0.05	≤0.05	≤1/19	≤1/19	≤0.05	>0.05	>0.05	>0.05	>0.05	>0.05	>0.05	≤0.05		>0.05	>0.05	≤0.05	>0.05	0.05	>0.05	>0.05	>0.05			>0.05				0.05	≤0.05	≤0.05	>0.05	≤0.05	≤0.05	≤0.05	>0.05
Trimethoprim	S susceptible					1																																		
	I intermediate																																							
	R resistant					8																	1	1				2												
	MIC 50					>4																	>4	>4				>4												
	MIC 90					>4																	>4	>4				>4												
Levofloxacin	S susceptible	1			12											1					1						1							1	1					
	I intermediate																																							
	R resistant	3		1	15									1	1	2				1		1		1	1	1										1		1		
	MIC 50	>2		>2	>2									>2	>2	>2				≤0.5	≤0.5	≤0.5		>2	>2	>2	≤0.5							≤0.5	≤0.5	>2		>4		
	MIC 90	>2		>2	>2									>2	>2	>2				≤0.5	≤0.5	≤0.5		>2	>2	>2	≤0.5							≤0.5	≤0.5	>2		>4		
Ciprofloxacin	S susceptible	1			12	4		2	4	1						1					1		1				1								1		1		1	
	I intermediate																																	1						
	R resistant	3		1	15	5	1	5	4		9	1	5	1	1	2		1	1	1		1		1	1	1		2	2				1			1				
	MIC 50	>1		>1	>1	>1	>4	>4	≤0.5	≤0.5	>4	>4	>4	>1	>1	>1		>4	>4	>1	≤0.25	1	≤0.25	>1	>1	>1	≤0.25	>1	>4				>4	0.5	≤0.25	>1	≤0.5		≤0.5	
	MIC 90	>1		>1	>1	>1	>4	>4	>4	≤0.5	>4	>4	>4	>1	>1	>1		>4	>4	>1	≤0.25	1	≤0.25	>1	>1	>1	≤0.25	>1	>4				>4	0.5	≤0.25	>1	≤0.5		≤0.5	
Moxifloxacin	S susceptible							2	4	1																			1								1		1	
	I intermediate																																							
	R resistant						1	5	4		9	1																	1				1					1		
	MIC 50						>1	>1	≤0.25	≤0.25	>1	>1																	≤0.25				>1				≤0.25	>2	≤0.25	
	MIC 90						>1	>1	>1	≤0.25	>1	>1																	>1				>1				≤0.25	>2	≤0.25	
Nitrofurantoin	S susceptible					8							7																											
	I intermediate																																							
	R resistant					1																		1	1			1												
	MIC 50					32							≤16											>64	>64			>64												
	MIC 90					32							≤16											>64	>64			>64												
Fusidic acid	S susceptible						1	5	8	1	7	1																	1				1				1		1	
	I intermediate																												1											
	R resistant							2			2		6				2	1	1																					
	MIC 50						≤0.5	≤0.5	≤0.5	≤0.5	≤0.5	≤0.5	4				≤0.5	≤0.5	4										≤0.5				≤0.5				≤0.5		≤0.5	
	MIC 90						≤0.5	2	≤0.5	≤0.5	8	≤0.5	8				8	≤0.5	4										8				≤0.5				≤0.5		≤0.5	
Mupirocin high level	S susceptible						1	6	7																															
	I intermediate																																							
	R resistant							1	1																															
	MIC 50						≤256	≤256	≤256																															
	MIC 90						≤256	≤256	≤256																															

**Amphoteri**	**$S^{susceptible}$**											1	1	2	1								
	**$I^{intermediate}$**																						
	**$R^{resistant}$**																						
	**MIC 50**											0.5	≤0.25	≤0.25	0.5								
	**MIC 90**											0.5	≤0.25	≤0.25	0.5								
**Casponfungi**	**$S^{susceptible}$**											1	1	1									
	**$I^{intermediate}$**													1									
	**$R^{resistant}$**														1								
	**MIC 50**											≤0.12	0.25	≤0.12	≥8								
	**MIC 90**											≤0.12	0.25	0.25	≥8								
**Flucytosin**	**$S^{susceptible}$**											1	1	2	1								
	**$I^{intermediate}$**																						
	**$R^{resistant}$**																						
	**MIC 50**											≤1	≤1	≤1	4								
	**MIC 90**											≤1	≤1	≤1	4								
**Fluconazo**	**$S^{susceptible}$**											1											
	**$I^{intermediate}$**																						
	**$R^{resistant}$**												1		1								
	**MIC 50**											≤0.5	16		8								
	**MIC 90**											≤0.5	16		8								
**Micafungi**	**$S^{susceptible}$**											1	1	2	1								
	**$I^{intermediate}$**																						
	**$R^{resistant}$**																						
	**MIC 50**											≤0.06	≤0.06	≤0.06	0.12								
	**MIC 90**											≤0.06	≤0.06	≤0.06	0.12								
**Voriconaz**	**$S^{susceptible}$**											1	1		1								
	**$I^{intermediate}$**													2									
	**$R^{resistant}$**																						
	**MIC 50**											≤0.12	≤0.12	0.25	0.25								
	**MIC 90**											≤0.12	≤0.12	0.25	0.25								

Table S2 Complete list of microorganisms and drugs tested for 2021

	MIC (µg/mL) Breakpoints	*Escherichia coli* N (%)=18 (16%)			*Klebsiella pneumoniae* N (%)= 15 (14%)				*Staphylococcus haemolyticus* N (%)= 14 (13%)				*Candida albicans* N (%)= 9 (8%)			*Staphylococcus aureus* N (%)= 6 (5%)				*Staphylococcus hominis* N (%)= 6 (5%)	*Staphylococcus epidermidis* N (%)= 6 (5%)	*Candida gabrata* N (%)= 5 (4%)			*Enterococcus faecalis* N (%)= 4 (4%)		*Pseudomonas aeruginosa* N (%)= 4 (4%)		*Enterococcus faecium* N (%)= 3 (3%)	
		EMO	*TOF*	*RET*	*EMO*	*TOF*	*RET*	*URO*	*EMO*	*TOF*	*NAS*	*ESP*	*EMO*	*TOF*	*URO*	*EMO*	*TOF*	*NAS*	*URO*	*EMO*	*EMO*	*EMO*	*TOF*	*NAS*	*EMO*	*URO*	*TOF*	*RET*	*EMO*	*TOF*
**Ampicillin**	S susceptible	1	2	2																					1	3				
	I intermediate																													
	R resistant	1		12	1	3	10	1	4	7	2	1				1	1	2		5	6						3	1	1	2
	**MIC 50**	≤2	>8	>8	>16	>16	>8	>8									>1	>1							≤2	≤2	>8	>8	>8	>8
	**MIC 90**	>8	>8	>8	>16	>16	>16	>8									>1	>1							≤2	≤2	>8	>8	>8	>8
**Oxacillin**	S susceptible															1				1	2									
	I intermediate																													
	R resistant								4	7	2	1					1	2		5	4									
	**MIC 50**								>2	>2	>2	>2				1	>2	>2		>2	>2									
	**MIC 90**								>2	>2	>2	>2				1	>2	>2		>2	>2									
**Penicillin G**	S susceptible																													
	I intermediate																													
	R resistant								4	7	2	1				1	1	2											1	
	**MIC 50**															>0.25	>0.25	>0.25											>0.25	
	**MIC 90**															>0.25	>0.25	>0.25											>0.25	
**Piperacillin**	S susceptible	1		2																							1			
	I intermediate																										2			
	R resistant	1		12			7	1																				1		1
	**MIC 50**	≤4		>16			>16	>16																			16	>16		
	**MIC 90**	>16		>16			>16	>16																			16	>16		
**Piperacillin-Tazobactam**	S susceptible	1	1	11	1	3	3																							
	I intermediate																										3			
	R resistant	1	1	3			7	1																				1		
	**MIC 50**	≤4/4	≤4/4	≤4/4	≤4/4	≤4/4	>16/4	>16/4																			16/4	>16/4		
	**MIC 90**	>16/4	>16/4	>16/4	≤4/4	≤4/4	>32/4	>16/4																			16/4	>16/4		

**Amoxicillin-clavulanate**	**S**susceptible	1		4	1		1																		
	**I**intermediate																								
	**R**resistant	1	1	5		3	7	1														3	1		
	**MIC 50**	4/2	>32/2	>32/2	≤2/2	>32	>32/2	>32/2														>32/2	>32/2		
	**MIC 90**	>32/2	>32/2	>32/2	≤2/2	>32	>32/2	>32/2														>32/2	>32/2		
**Cefalexine**	**S**susceptible																								
	**I**intermediate																								
	**R**resistant																								
	**MIC 50**																								
	**MIC 90**																								
**Cefuroxime**	**S**susceptible		1	7	1		1																		
	**I**intermediate	2		2																					
	**R**resistant		1	5		3	9	1														3	1		1
	**MIC 50**	4	4	4	≤4	≤4	>8	>8														>8	>8		
	**MIC 90**	4	>8	>8	≤4	≤4	>16	>8														>8	>8		
**Cefoxitine**	**S**susceptible																								
	**I**intermediate																								
	**R**resistant								3	6	2	1			1	2	5	3		1	2			1	2
	**MIC 50**								>8	>8	>8	>8			>8	>8	>8	>8		>8	>8			>8	>8
	**MIC 90**								>8	>8	>8	>8			>8	>8	>8	>8		>8	>8			>8	>8
**Ceftazidime**	**S**susceptible	2	1	11	1		2																1		
	**I**intermediate																					3			
	**R**resistant		1	3		3	8	1																1	1
	**MIC 50**	≤0.5	≤0.5	≤0.5	≤1	>16	>8	>8														8	≤0.5	≤0.25	
	**MIC 90**	≤0.5	>8	>8	≤1	>16	>16	>8														8	≤0.5	≤0.25	
**Cefotaxime**	**S**susceptible	2	1	9	1	3	2																		
	**I**intermediate																								
	**R**resistant		1	5			8	1														3			1
	**MIC 50**	≤1	≤1	≤1	≤1	≤1	>4	>4														>4			
	**MIC 90**	≤1	>4	>4	≤1	≤1	>4	>4														>4			
**Cefixime**	**S**susceptible																								
	**I**intermediate																								
	**R**resistant																								
	**MIC 50**																								
	**MIC 90**																								
**Cefepime**	**S**susceptible	2		6	1																		1		
	**I**intermediate																					2			
	**R**resistant		2	8		3	10	1														1			1
	**MIC 50**	≤1	>8	8	≤1	>16	>8	>8														8	8		
	**MIC 90**	≤1	>8	>8	≤1	>16	>16	>8														8	8		
**Ceftaroline**	**S**susceptible													1											
	**I**intermediate																								
	**R**resistant																			1	3				2
	**MIC 50**													0.5											>1
	**MIC 90**													0.5											>1

Ertapenem Ertapenem	$S^{susceptible}$	1			1		1																			
	$I^{intermediate}$																									
	$R^{resistant}$	1	2	14		3	9	1															3	1		
	MIC 50		>1	>1	≤0.25	>2	>1	>1															>1	>1		
	MIC 90	>1	>1	>1	≤0.25	>2	>2	>1															>1	>1		
Imipenem	$S^{susceptible}$	2	1	7	1	3	2											1	2		1	2	1			
	$I^{intermediate}$						1															1	2			
	$R^{resistant}$		1	7			6	1	4	6	2	1			1	2		4	4					1	1	2
	MIC 50	≤0.25	≤0.25	2	≤0.25	≤0.25	>8	>8	>8	>8	>8	>8			>8	>8		≤2	4		≤2	≤2	1	8	>8	>8
	MIC 90	1	>8	>8	≤0.25	≤0.25	>8	>8	>8	>8	>8	>8			>8	>8		>8	>8		≤2	>4	2	8	>8	>8
Meropenem	$S^{susceptible}$	2		2	1		1																3			
	$I^{intermediate}$			5			1																			
	$R^{resistant}$		2	7		3	8	1																1		
	MIC 50	≤0.125	>8	8	≤0.125	>8	>8	>8															2	>8		
	MIC 90	2	>8	>8	≤0.125	>8	>8	>8															2	>8		
Vancomicin	$S^{susceptible}$								3	6	2	1		1		2		3	5		1	2			1	1
	$I^{intermediate}$																									
	$R^{resistant}$								1	1					1			3	1			1				1
	MIC 50								4	2	2	2		1	≤0.5	≤0.5		≤0.5	2		2	1			1	≤0.5
	MIC 90								8	2	2	2		1	≤0.5	2		8	>8		2	>8			1	>8
Fosfomycin	$S^{susceptible}$	2	1	11	1	3	2	1	2	4	1	1		1	1	2	1	3	5				1			
	$I^{intermediate}$																									
	$R^{resistant}$		1	3			8		2	3	1					1		3	1							
	MIC 50	≤16	≤16	≤16	32	≤16	>64	≤16	>64	32	≤16	≤16		≤16	≤16	≤16	≤16	32	≤16				32			
	MIC 90	≤16	>64	>64	32	≤16	>64	≤16	>64	64	>64	≤16		≤16	≤16	≤16	≤16	>64	64				32			
Daptomycin	$S^{susceptible}$								3	7	2	1		1	1	3	1	6	5			1				
	$I^{intermediate}$																									
	$R^{resistant}$								1										1							
	MIC 50								≤0.5	≤0.5	≤0.5	≤0.5		1	≤0.5	≤0.5	≤0.5	≤0.5	≤0.5			>4				
	MIC 90								2	≤0.5	≤0.5	≤0.5		1	≤0.5	≤0.5	≤0.5	≤0.5	>4			>4				
Teicoplanin	$S^{susceptible}$								3	6	1	1			1	2	1	4	5		1	2			1	1
	$I^{intermediate}$																									
	$R^{resistant}$								1	1	1					1		2	1			1				1
	MIC 50								4	4	4	4			≤0.5	≤0.5	≤0.5	≤0.5	4		≤0.5	≤0.5			1	≤0.5
	MIC 90								>8	4	8	4			≤0.5	4	≤0.5	>8	>8		≤0.5	>8			1	>8
Colistin	$S^{susceptible}$			1																						
	$I^{intermediate}$																									
	$R^{resistant}$		1	3																						1
	MIC 50		>4	>4																						
	MIC 90		>4	>4																						
Linezolid	$S^{susceptible}$								4	7	2	1		1	1	2		5	5		1	2			1	2
	$I^{intermediate}$																									
	$R^{resistant}$																	1	1			1				
	MIC 50								2	1	1	1		4	2	1		2	2		1	1			2	2
	MIC 90								2	2	2	1		4	2	2		2	>4		1	>4			2	2

Cloramphenicol	$S^{susceptible}$																									
	$I^{intermediate}$																									
	$R^{resistant}$																									
	MIC 50																									
	MIC 90																									
Erythromycin	$S^{susceptible}$														1	1	1			2						
	$I^{intermediate}$																									
	$R^{resistant}$								4	7	2	1				1		6	4		1	2			1	1
	MIC 50								>2	>2	>2	>2		0.5	≤0.25	≤0.25		>2	>2		>2	1			>2	>2
	MIC 90								>2	>2	>2	>2		0.5	≤0.25	>2		>2	>2		>2	1			>2	>2
Clindamycin	$S^{susceptible}$								1	2	1	1		1	1	2	1	2	2							
	$I^{intermediate}$																	1								
	$R^{resistant}$								3	5	1					1		3	4		1	3				2
	MIC 50								>1	≤0.25	≤0.25	≤0.25		≤0.25	≤0.25	≤0.25	≤0.25	0.5	>1		>1	>1				≤0.25
	MIC 90								>1	>1	>1	≤0.25		≤0.25	≤0.25	≤0.5	≤0.25	>1	>1		>1	>1				>1
Amikacin	$S^{susceptible}$	2	1	12	1	1	8																3	1		
	$I^{intermediate}$			1																						
	$R^{resistant}$		1	1			2	1																		1
	MIC 50	≤4	≤4	≤4	≤4	≤4	≤4	≤4															≤4	≤4		
	MIC 90	≤4	>16	≤4	≤4	≤4	>16	≤4															≤4	≤4		
Gentamicin	$S^{susceptible}$	2	1	12	1	3	5							1	1	2	1	2	3				1	1		
	$I^{intermediate}$																									
	$R^{resistant}$		1	2			5	1	4	7	2	1				1		4	3		1	1			1	2
	MIC 50	≤1	≤1	≤1	≤1	≤1	>4	>4	>4	>4	>4	>4		≤1	≤1	≤1	≤1	2	>4		>4	>4	≤1	≤1	>4	>4
	MIC 90	≤1	>4	>4	≤1	≤1	>4	>4	>4	>4	>4	>4		≤1	≤1	>4	≤1	>4	>4		>4	>4	≤1	≤1	>4	>4
Tobramycin	$S^{susceptible}$	2	1	11	1	3	5																3	1		
	$I^{intermediate}$																									
	$R^{resistant}$		1	3			5	1																		1
	MIC 50	≤1	≤1	≤1	≤2	≤2	≤2	>4															≤1	≤1		
	MIC 90	2	>4	>4	≤2	≤2	8	>4															2	≤1		
Tetracycline	$S^{susceptible}$									2	1	1		1		1	1	3	4							
	$I^{intermediate}$									4																
	$R^{resistant}$								4	1	1				1	2		3	2							
	MIC 50								>2	2	≤0.5	≤0.5		≤0.5	>2	>2	≤0.5	≤0.5	1							
	MIC 90								>2	2	>2	≤0.5		≤0.5	>2	>2	≤0.5	>2	>2							
Tigecycline	$S^{susceptible}$	2	2	13					3	7	2	1		1	1	3	1	6	5		1					
	$I^{intermediate}$																									
	$R^{resistant}$			1					1										1				3	1		
	MIC 50	≤0.5	≤0.5	≤0.5					≤0.25	≤0.25	≤0.25	≤0.25		≤0.25	≤0.5	≤0.25	≤0.25	≤0.25	≤0.25		≤0.25		>2	≤0.5		
	MIC 90	≤0.5	≤0.5	≤0.5					1	≤0.25	≤0.25	≤0.25		≤0.25	≤0.5	0.5	≤0.25	≤0.25	≤0.25		≤0.25		>2	≤0.5		
Rifampicin	$S^{susceptible}$																		1							
	$I^{intermediate}$									1																
	$R^{resistant}$								2	4	1								2							
	MIC 50								>1	>1	>1								0.5							
	MIC 90								>1	>1	>1								>1							

Trimethoprim-sulfamethoxaz	$S^{susceptible}$	2	2	10	1	3	4										1	3	1	2	4									
	$I^{intermediate}$																			1										
	$R^{resistant}$			4			6	1	4	7	2	1				1				3	2				1	1	1			1
	MIC 50	≤1/19	≤1/19	≤1/19	≤1/19	≤1/19	>4/76	>4/76	>4/76	>4/76	>4/76	>4/76				>4/76	≤1/19	≤1/19	≤1/19	4/76	≤1/19				≤1/19	≤1/19	>4/76			≤1/19
	MIC 90	≤1/19	≤1/19	>4/76	≤1/19	≤1/19	>4/76	>4/76	>4/76	>4/76	>4/76	>4/76				>4/76	≤1/19	≤1/19	≤1/19	>4/76	>4/76				≤1/19	≤1/19	>4/76			≤1/19
Trimethoprim	$S^{susceptible}$																													
	$I^{intermediate}$																													
	$R^{resistant}$																													
	MIC 50																													
	MIC 90																													
Levofloxacin	$S^{susceptible}$	1	1	8	1	3	2																							
	$I^{intermediate}$			1																							1			
	$R^{resistant}$	1	1	5			8	1																			2	1		
	MIC 50	≤0.5	≤0.5	≤0.5	≤0.5	≤0.5	>2	>2																			>2	>2		
	MIC 90	>2	>2	>2	≤0.5	≤0.5	>8	>2																			>2	>2		
Ciprofloxacin	$S^{susceptible}$	1	1	6	1	3	2											1	1	1	1					2	1			
	$I^{intermediate}$			2												1				2							1			
	$R^{resistant}$	1	1	5			8	1	4	7	2	1					1	2		3	5					1	1	1		2
	MIC 50	≤0.25	≤0.25	≤0.25	≤0.125	≤0.125	>1	>1	>4	>4	>4	>4				1	>4	>4	≤0.5	≤0.5	>4					≤0.5	0.5	>1		>4
	MIC 90	>1	>1	>1	≤0.125	≤0.125	>1	>1	>4	>4	>4	>4				1	>4	>4	≤0.5	>4	>4					4	1	>1		>4
Moxifloxacin	$S^{susceptible}$															1		1	1	3	1									
	$I^{intermediate}$																													
	$R^{resistant}$								4	7	2	1					1	2		3	5									
	MIC 50								>1	>1	>1	>1				≤0.25	>1	>1	≤0.25	≤0.25	>1									
	MIC 90								>1	>1	>1	>1				≤0.25	>1	>1	≤0.25	>1	>1									
Nitrofurantoin	$S^{susceptible}$																													
	$I^{intermediate}$																													
	$R^{resistant}$																													
	MIC 50																													
	MIC 90																													
Fusidic acid	$S^{susceptible}$								3	7	2	1				1	1	2		6	3									
	$I^{intermediate}$																													
	$R^{resistant}$								1												3				1	2			1	1
	MIC 50								≤0.5	≤0.5	≤0.5	≤0.5				≤0.5	≤0.5	≤0.5		≤0.5	4				4	2			8	4
	MIC 90								4	≤0.5	≤0.5	≤0.5				≤0.5	≤0.5	≤0.5		≤0.5	>8				4	2			8	4
Mupirocin high level	$S^{susceptible}$															1	1	3												
	$I^{intermediate}$																													
	$R^{resistant}$																													
	MIC 50															≤256	≤256	≤256												
	MIC 90															≤256	≤256	≤256												
ANFOTERACINA B	$S^{susceptible}$												1	6	1							1	3	1						
	$I^{intermediate}$													1																
	$R^{resistant}$																													
	MIC 50												0.5	1	0.5							1	≤0.25	≤0.25						
	MIC 90												0.5	1	0.5							1	0.5	≤0.25						

CASPOFUNGINA	$\text{S}^{\text{susceptible}}$				1 6 1							
	$\text{I}^{\text{intermediate}}$											
	$\text{R}^{\text{resistant}}$				1				1 3 1			
	**MIC 50**				≤0.12 ≤0.12 ≤0.12				0.5 0.5 0.5			
	**MIC 90**				≤0.12 0.25 ≤0.12				0.5 0.5 0.5			
FLUCITOSINA	$\text{S}^{\text{susceptible}}$				1 7 1				1 3 1			
	$\text{I}^{\text{intermediate}}$											
	$\text{R}^{\text{resistant}}$											
	**MIC 50**				≤1 ≤1 ≤1				≤1 ≤1 ≤1			
	**MIC 90**				≤1 ≤1 ≤1				≤1 ≤1 ≤1			
FLUCONAZOLO	$\text{S}^{\text{susceptible}}$				1 6 1							
	$\text{I}^{\text{intermediate}}$				1							
	$\text{R}^{\text{resistant}}$											
	**MIC 50**				1 1 1							
	**MIC 90**				1 1 1							
MICAFUNGINA	$\text{S}^{\text{susceptible}}$				1 6 1				1 3 1			
	$\text{I}^{\text{intermediate}}$											
	$\text{R}^{\text{resistant}}$				1							
	**MIC 50**				≤0.06 ≤0.06 ≤0.06				≤0.06 ≤0.06 ≤0.06			
	**MIC 90**				≤0.06 ≤0.06 ≤0.06				≤0.06 ≤0.06 ≤0.06			
VORICONAZOLO	$\text{S}^{\text{susceptible}}$				1 7 1				1 2 1			
	$\text{I}^{\text{intermediate}}$											
	$\text{R}^{\text{resistant}}$											
	**MIC 50**				≤0.12 ≤0.12 ≤0.12				≤0.12 ≤0.12 0.25			
	**MIC 90**				≤0.12 ≤0.12 ≤0.12				≤0.12 ≤0.12 0.25			
